# Face Mask Reduces the Effect of Proposer’s (Un)Trustworthiness on Intertemporal and Risky Choices

**DOI:** 10.3389/fpsyg.2022.926520

**Published:** 2022-06-16

**Authors:** Loreta Cannito, Stefano Anzani, Alessandro Bortolotti, Alberto Di Domenico, Riccardo Palumbo

**Affiliations:** ^1^Department of Psychological Sciences, Health and Territory (DiSpuTer), University G. d’Annunzio of Chieti-Pescara, Chieti Scalo, Italy; ^2^Center for Advanced Studies and Technology (CAST), University “G. d’Annunzio” of Chieti-Pescara, Chieti Scalo, Italy; ^3^Department of Neuroscience, Imaging and Clinical Sciences (DNISC), University G. d’Annunzio of Chieti-Pescara, Chieti Scalo, Italy

**Keywords:** face mask, perceived trustworthiness, delay discounting, probability discounting, risk taking

## Abstract

Previous literature suggested that individuals increase temporal and risk discounting at the presence of a proposer whose face is perceived as untrustworthy, suggesting the activation of protective choice patterns. By the way, the COVID-19 pandemic has substantially transformed the way we interact with other people, even bringing us into situations where the face of the person making a proposal is not fully visible, because of the mask. With the current study, we aimed at verifying if the effect of proposer’s facial (un)trustworthiness on discounting behavior is modulated by mask wearing. In two different experiments, participants performed traditional delay and probability discounting tasks with masked proposers manipulated across trustworthiness levels. Results highlighted that, even after checking for subject-specific emotion recognition ability with masked faces, the presence of a masked untrustworthy proposer increases both delay and probability discounting parameters, although the effect is not statistically significant and smaller than the one detected at the presence of an untrustworthy proposer without a mask. These results suggest that the ability to perceive the proposer’s (un)trustworthiness is affected by the mask, with a consequent less strong effect of proposer’s (un)trustworthiness on choice behavior on both intertemporal and risky choices. Limits and possible implications are outlined and discussed.

## Introduction

### Trustworthiness and Discounted Choices

In recent years, research on variables influencing decision-making started to devote more attention to the investigation of the role of social factors and actors in this domain. Particularly, when considering models of discounted decision-making, some evidence has been collected on the differences on decisional outcomes, due to decision-maker’s individual propensity to trust others as well as on the role of proposer’s perceived trustworthiness. In our everyday life, when faced with someone making a proposal and asking us for a choice between possible courses of action, visible features of the proposer can play a crucial role. Children, for example, have been shown to enhance their willingness to wait in order to get a more attractive reward, as assessed *via* traditional marshmallow test, when the person proposing the choice and delivering the incentive is regarded as trustworthy, as based on both, face appearance (Michaelson et al., 2013) and observed behavior during previous interactions (Michaelson and Munakata, 2016). Of relevance, effect of trustworthiness may act also without previous direct knowledge of proposer trustworthiness and by the means of reputational influence (e.g., Izuma, 2012; Ponsi et al., 2017; Bellucci and Park, 2020). Trust-based factors have been found to play a role in postponing gratification even when children had no knowledge about the individual providing the future reward (neither face nor behavior), since simply having a greater degree of generalized trust in people led children to wait longer (Ma et al., 2018). A possible explanation for this result with untrustworthy proposer has been linked to the waiting time typically included in temporal discounting protocols, such as that, as based on deliberative reasoning process, people tend to prefer immediate rewards as the delayed one is perceived as not surely obtainable in future, given the proposer’s untrustworthiness. Also, others’ perceived untrustworthiness can also feel like a danger, triggering unpleasant feelings that impact intertemporal and risk decisions (Harris, 2012; Koppel et al., 2017). By the way, in a recent paper on adult samples, it was not only reported that proposer’s facial perceived untrustworthiness is associated with higher temporal discounting rate therefore indicating lower preference toward reward’s postponing, but also that the same effect applies to subjects’ probability discounting, for which participants were asked to choose between smaller sure and larger but risky options and were aware that reward delivery system was based on randomness (Anzani et al., 2022). This evidence indicating a lower propensity toward risk taking with untrustworthy proposers, even if in need of replication, seems to suggest the possibility that this effect of untrustworthiness on decision-making may be sustained by a more domain-independent and less deliberative underlying process than ever thought before. Despite specific mechanisms involved in this phenomenon, taken together, evidence accumulated in literature until now, seem to suggest a crucial role of proposer’s (perceived) (un)trustworthiness in our everyday choice outcomes, particularly when considering face-to-face interactions.

### Impact of Mask Wearing

The COVID-19 pandemic that hit the world in the 2020 has caused drastic changes in our usual habits both on personal (e.g., Cannito et al., 2022) and societal level (e.g., Ceccato et al., 2021). In order to contain the virus spread, several restraint measures have been introduced, such as avoiding direct contact with other people and wearing masks. While existing evidence suggest that some processes, such as social attention, are not significantly affected by mask wearing (e.g., Dalmaso et al., 2021), both reduction of interpersonal interaction and impossibility to access to the whole set of facial expressions have the potential to produce an influence on face-to-face interactions and to affect social relationships (e.g., Carbon, 2020). For instance, some studies already shown that wearing face mask reduces accuracy in emotion recognition and perceived closeness (Grundmann et al., 2021) and that this effect is even larger for individuals with autistic traits (Pazhoohi et al., 2021). Moreover, a reduction in accuracy when identifying emotions in masked vs. no-masked faces stimuli was also reported for a population, healthcare students, that is planned to be exposed to masked human faces in the next future (Bani et al., 2021) and across the lifespan with older adults (Schroeter et al., 2021), adults (e.g., Carbon, 2020), and children (Ruba and Pollak, 2020) experiencing the same effect. While generally agreeing on the phenomenon, existing results in literature highlight some differences for what concerns the expressed emotion. By way of illustration, some evidence report that this effect is present for all the basic tested emotions (e.g., Pazhoohi et al., 2021) while other report that this does not apply to some emotions, such as fear (e.g., Carbon, 2020) or for neutral expression (e.g., Marini et al., 2021). Furthermore, some evidence highlighted that the effect of mask wearing influences not only emotion recognition but also other face-induced perceived features, such as perceived trustworthiness. For example, it was recently shown that a masked face received significantly lower perceived trustworthiness evaluations as compared to the no-masked version (Gabrieli and Esposito, 2021). Similarly, in another work, authors reported a similar result also showing that reduced trustworthiness effect for masked stimuli was stronger for those participants who thought that mask had a poorer protecting capability and felt more burdened when wearing it (Biermann et al., 2021). Following this line of reasoning, it can be hypothesized that when faced with an (already) untrustworthy proposer wearing a mask, an augmented effect on decision-maker’s discounting behavior should be detected (i.e., a higher shift toward immediate and sure options as compared to results reported by Anzani et al., 2022). On the other side, it was recently shown that wearing mask affects other perceived features, for example, it increases perceived attractiveness for both male and female stimuli (Hies and Lewis, 2022; Parada-Fernández et al., 2022). Therefore, as attractiveness have been reported to increase perceived trustworthiness (Pandey and Zayas, 2021) and trustworthiness have been proved to influence decision-making outcomes (e.g., Jaeger et al., 2019; Qi et al., 2021; Anzani et al., 2022), it can also be hypothesized that, through an indirect effect due to increased perceived attractiveness (already) untrustworthiness proposers are perceived as less untrustworthy and, therefore, a reduced effect on decision-maker’s discounting behavior should be detected. To deeper explore this phenomenon and investigate whether the presence of surgical mask produce a change on the effect of proposer’s trustworthiness on decision-making and to which extent, in the current work, we replicated the experimental procedure proposed by Anzani et al. (2022) and conducted two separate experiments (experiment A investigating delay discounting and experiment B investigating probability discounting) after manipulating proposer’s stimuli to which a face surgical mask was applied.

## Materials and Methods

### Sample

The sample was composed of 43 volunteers (19 male, mean age = 27.0, SD = 8.8 years) who performed the experimental procedure in experiment A and 45 volunteers (23 male, mean age = 20.1, SD = 1.9 years) who performed in experiment B. All participants were neurotypical and had no psychiatric or addiction history. We decided to screen participants with the characteristic as each of them has been proved to influence, in different ways, discounting behavior (e.g., Amlung et al., 2019; Mok et al., 2021). The experiment was performed online, with the platform E-primeGO (Psychology Software Tools, Inc. E-Prime Go; 2020).^1^ All participants from both experiments received a link *via* email through which they were presented a request for informed consent to take part in the study, together with initial instructions about the tasks to be performed. Once they had given their consent, participants were directed to the experimental procedure. Data were collected during November 2020, with concomitant data gathering schedule for the two experiments, to avoid setting manipulation differences due to time of administration. Participants received no money or other form of compensation to take part in this study. The study complies with the Declaration of Helsinki and received approval from the reference Ethics Committee.

### Behavioral Task

Before the task administration, participants were asked to answer a brief survey including demographic questions about some basic information, such as their age and gender, and clinical history as screening criteria. No participants were excluded as based on these questions. After recruiting, participants have been randomly assigned to experiment A (delay discounting) or to experiment B (probability discounting).

**Figure 1:**
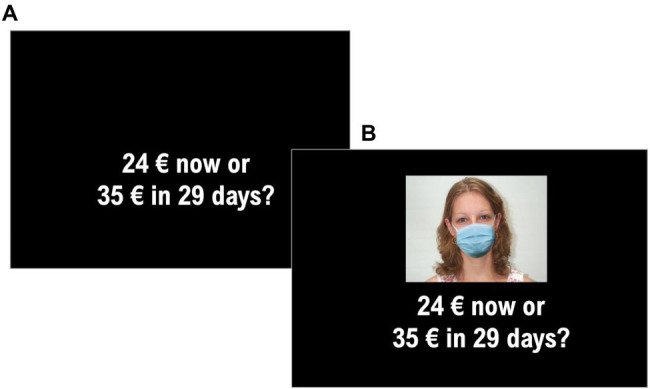
Trial example in the “baseline” **(A)** and “proposer” block **(B)**.

**Figure 2:**
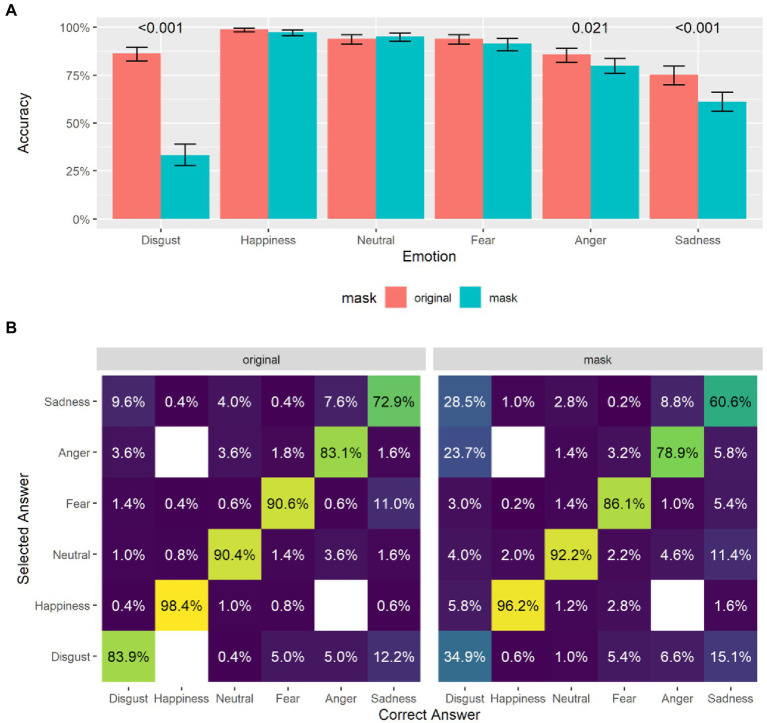
**(A)** Emotion recognition accuracy across emotions and stimuli conditions. (**B)** Confusion matrix on emotion recognition.

For both studies, participants were presented choice items from the Money Choice Questionnaire (MCQ, experiment A) or the Probability Discounting Questionnaire (PDQ, experiment B), which consisted of the standard delay and probability discounting questionnaires from Kirby et al. (1999) and Madden et al. (2009), respectively, in seven different blocks (1 baseline block and 6 “proposer” blocks). In the baseline block, participants were standardly presented the two choice options and asked to choose as quickly as possible using the keyboard, pressing “A” and “L” keys. For the MCQ, participants were asked to choose between smaller sooner option and larger delayed options, respectively. Half of the items required an inverted response system (“A” for larger and “L” for smaller) in order to avoid side bias. Same approach was employed for the PDQ at which participants were asked to choose between a smaller sure option and larger probabilistic one. For both task, participants were also presented choice items in “proposer” blocks, which provided a face stimulus and were asked to imagine that the showed face was the person proposing the choice between the two options while having no role in potential money delivery (see Figure 1). The used facial stimuli were manipulated for gender (male and female) and for perceived trustworthiness level (trustworthy, neutral, and untrustworthy) resulting in six different blocks (for a similar procedure, see Anzani et al., 2022). Order of presentation of the seven blocks was randomized as well as items’ order within each block. In order to create the masked versions of the face stimuli in the proposer blocks, the original pictures from Minear and Park (2004) were edited with open-source graphical manipulation software GIMP (version 2.10.22). The mask was adapted to the best fit to each face, and colors and shadows were matched to ensure a realistic rendering of the pictures. Each item of the questionnaire was shown with all six masked proposers, so the whole experiment consisted in 27 (baseline) + 162 trials (proposer blocks) for the MCQ and 30 (baseline) + 180 trials (proposer blocks) for the PDQ. Also, for both studies, participants completed a second behavioral task investigating participants’ ability to recognize emotions expressed by facial masked stimuli. To this purpose, a total of 36 stimuli were obtained from FACES database (Ebner et al., 2010). Selected stimuli varied across gender (male and female), age (young adult, adult, and elderly) and expressed emotion (neutral, sadness, happiness, disgust, fear, and anger). Then, a modified version of each stimulus was created by adding a fitted mask (see Figure 2 for stimuli example). Stimulus editing was performed *via* GIMP (version 2.10.22). For both studies, half of participants performed emotion recognition task as first and discounting task (MCQ or PDQ) as second, while the other half performed the tasks in reverse order.

### Data Pre-processing and Analysis

One participant who performed the MCQ (experiment A) and four participants who performed the PDQ (experiment B) were discarded from subsequent analysis because their data were indistinguishable from random choices. To determine this, we compared the percentage of correct answers to the emotion recognition task of each participant and considered random responders those who had an accuracy comparable to random chance (1 over 6 = 16%). Therefore, the final sample was composed of 42 participants for experiment A and 41 participants for experiment B. Data pre-processing and statistical analysis were carried out using R. Computation of discounting parameters for both tasks and for each block was based on the R syntax developed by Gray et al. (2016). Starting from participants’ observed preferences, the syntax allows to calculate the discounting parameters (*k* and *h*) by assigning the most coherent parameter taking it from a pre-compiled list based on the hyperbolic discounting models (Mazur, 1987). The parameters come from the following two equations, where V is the subjective value of the reward A after a delay T:


(1) $$V=\frac{A}{1+kT}$$

or with odds against winning $\Theta =\left(1-p\right)/p$ where p is the probability shown with the uncertain option:


(2) $$V=\frac{A}{1+h\Theta }$$

Obtained discounting parameters were then log-transformed to ensure normality as based on literature (e.g., Calluso et al., 2020; Cannito et al., 2021; Iodice et al., 2022).

Together with explicit preferences during the task, participants’ response times (RTs) in milliseconds (ms) were also collected. RTs data cleaning was performed by removing upper outliers at three standard deviations (SD) and excluding trial shorter than 250 ms. RTs were then re-scaled in seconds (s) to help with convergence of the mixed models used in the analysis.

All the analysis were carried out using mixed effects models with the *lme4* R package (Bates et al., 2015), omnibus tests were obtained with the *Anova* function from the *car* package (Fox and Weisberg, 2019), and *post-hoc* comparison and estimated marginal means were computed with the *emmeans* package (Lenth, 2021).

## Results – Emotion Recognition Task

As the effect of mask on discounting task may have been influenced by subject-specific ability to read facial cues from masked faces, participants’ ability to accurately recognize emotion as expressed by a masked face was assessed. A general linear mixed model was performed to test the effect of mask (mask and normal), of emotion (neutral, happiness, disgust, fear, anger, and sadness) and task (td and pd) on accuracy performance. Results highlighted no effect of task, but significant effect of mask, emotion, and interaction effect between the two (see Table 1; Figure 2A) with disgust (*p* < 0.001), sadness stimuli (*p* < 0.001), and anger stimuli (*p* = 0.02) showing a statistically significant difference in accuracy between mask and normal stimuli. We then conducted a more exploratory, qualitative error analysis using a confusion matrix, a double-entry table in which the columns indicate the expected (correct) responses, and the rows indicate the responses given by the participant. In this format, correct responses are placed along the diagonal of the matrix, and the other cells in each column indicate how the incorrect responses are distributed for each stimulus. In fact, the percentages reported are calculated per column (Figure 2B). The confusion matrix seems to suggest that, also with the original no masked stimuli, there was some confusion between expressions of disgust, sadness an anger, and that this confusion is greatly amplified with mask stimuli. After exploring participants’ performance on emotion recognition task across conditions, to test the possible role of subject-specific ability to recognize masked emotions on discounting behavior with (un)trustworthy masked proposers, participants’ random slope with mask stimuli in the emotion recognition task was entered in the following analyses on the effect of masked proposers with different levels of trustworthiness on delay (experiment A) and probability discounting (experiment B).

**Table 1:** Omnibus test for effects of mask, emotion, and task on emotion recognition accuracy.

	*χ* ^2^	DF	*p*
(Intercept)	**660.03**	1	**<0.001**
Mask	**40.94**	1	**<0.001**
Emotion	**326.65**	5	**<0.001**
Task	0.19	1	0.664
Mask: emotion	**140.63**	5	**<0.001**

Significant *effects* are reported in bold.

### Results Experiment A

#### Delay Discounting

**Figure 3:**
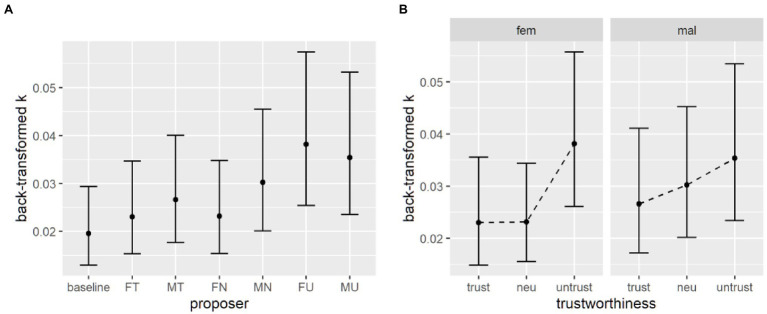
Discounting parameters back-transformed from the log space. **(A)** Parameter estimates from the first model. (**B)** Parameter estimates from the second model without the baseline.

Following previous literature (e.g., Calluso et al., 2019), delay discounting parameter k were log-transformed to address normality. To test for the effect of the proposer on delay discounting parameter’s change compared to baseline, we used a linear mixed effect model, with the current proposer as predictor of the log-transformed *k* value, and with a random intercept for each participant, accounting for individual differences and for the repeated measures design. The full code for all the models is available in the online repository. The model uses treatment coding for factors, with the baseline set as the reference value, so each coefficient of the model can be used to test the change in k due to each proposer. We apply the *t* as z criterion for significance of the coefficients, so *t* values higher than 2 can be considered significant. Even though this method has been shown to be anti-conservative, this mostly apply to smaller sample sizes (Luke, 2017). Results are reported in Table 2. Both male and female untrustworthy proposers and also the male neutral proposer elicit a significant increase in discounting rate compared to the baseline, indicating participants’ reduced availability to wait in order to obtain a larger reward (Figure 3A).

**Table 2:** Fixed effects of proposers compared to baseline.

				CI 95%	
Term	Estimate	SE	Statistic		
				LL	UL
(Intercept)	−3.936	0.204	**−19.277**	−4.336	−3.536
Proposer FT	0.165	0.179	0.920	−0.186	0.515
Proposer MT	0.310	0.179	1.732	−0.041	0.661
Proposer FN	0.170	0.179	0.951	−0.181	0.521
Proposer MN	0.437	0.179	**2.444**	0.087	0.788
Proposer FU	0.671	0.179	**3.748**	0.320	1.021
Proposer MU	0.594	0.179	**3.321**	0.244	0.945

*FT, female trustworthy; MT, male trustworthy; FN, female neutral; MN, male neutral; FU, female untrustworthy; MU, male untrustworthy; CI, confidence interval, LL, lower level; UL, upper level. Same labels are valid for all the subsequent tables. Significant effects are reported in bold*.

To test the different contributions of the proposer’s features, we used a second mixed effect model with gender and trustworthiness of the proposer as fixed factors and a random intercept for each subject and excluding the data coming from the baseline condition (for which the tested factors are meaningless). Proper omnibus tests for main effects and interactions were obtained by setting contrasts as sum contrasts (Singmann and Kellen, 2019). Results of omnibus tests (type 3, Wald *χ*^2^ tests) revealed that neither gender nor the level of trustworthiness of the proposer had a significant effect on the rate at which participants discount delayed options (see Supplementary Table S1; Figure 3B).

#### Response Times

**Figure 4:**
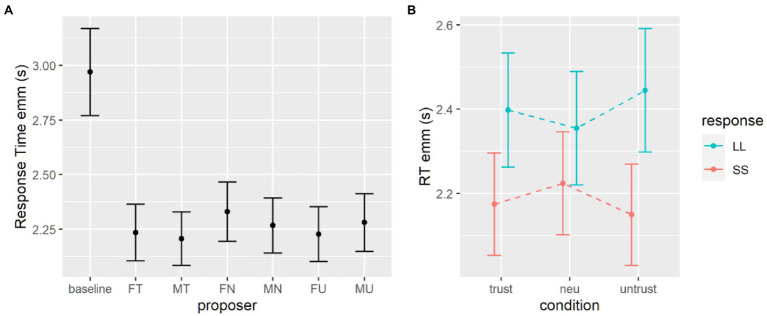
Estimates for response times in the delay discounting task with masked proposers. **(A)** Estimates from the first model, with baseline data. **(B)** Estimates from the second model, without the baseline data. SS, smaller sooner option; LL, larger later option.

For each subject only RTs higher than 250 ms and lower than three standard deviations over the mean were computed for that subject. The same generalized mixed effect model was employed to investigate response times (RTs). We set the family of the distribution to inverse Gaussian and coded the model with current proposer as fixed effect, and with a random intercept and slope for each subject (see Table 3). Results highlighted that all proposer conditions elicited mean RTs significantly faster than the baseline condition (Figure 4A).

**Table 3:** Fixed effects of proposers compared to baseline on RTs in the delay discounting task.

				CI 95%	
Term	Estimate	SE	Statistic		
				LL	UL
(Intercept)	2.990	0.102	**29.233**	2.789	3.190
Proposer FT	−0.723	0.104	**−6.953**	−0.926	−0.519
Proposer MT	−0.758	0.108	**−7.009**	−0.970	−0.546
Proposer FN	−0.629	0.099	**−6.333**	−0.824	−0.435
Proposer MN	−0.697	0.101	**−6.922**	−0.895	−0.500
Proposer FU	−0.733	0.096	**−7.615**	−0.922	−0.544
Proposer MU	−0.685	0.107	**−6.400**	−0.894	−0.475

*Significant results are in bold.*

We tested the effects of gender, level of trustworthiness and given response using the second generalized mixed effects model. Omnibus Wald tests show that the main effect of response is significant, participants were faster when expressing a preference for the immediate option (see Table 4). The two-way interaction between level of trustworthiness and given response is significant and looking at *post-hoc* comparisons we can see that the difference in response times between immediate and delayed options holds with trustworthy and untrustworthy proposers but is less stronger for neutral proposers (see Table 5; Figure 4B).

**Table 4:** Omnibus test of effects for gender, trustworthiness and given response on RTs.

	*χ* ^2^	DF	*p*
(Intercept)	**1,482.72**	1	**<0.001**
Gender	0.10	1	0.752
Trustworthiness level	0.03	2	0.985
Response	**61.08**	1	**<0.001**
Gender: trustworthiness level	3.42	2	0.181
Gender: response	0.20	1	0.652
Trustworthiness level: response	**6.63**	2	**0.036**
Gender: trustworthiness level: response	0.48	2	0.785

*Significant results are in bold.*

**Table 5:** *Post-hoc* comparison of RTs between given response within each level of trustworthiness.

Response contrast	Proposer	Estimate	SE	*p*
SS – LL	Trustworthy	−0.229	0.046	**<0.001**
SS – LL	Neutral	−0.131	0.046	**0.004**
SS – LL	Untrustworthy	−0.309	0.054	**<0.001**

*SS, smaller sooner option; LL, larger later option.*Significant results are in bold.

#### The Impact of Emotion Recognition Ability on *k* Parameter

As anticipated, for the analyses on the effect of proposers on discounting rate, we wanted to investigate whether participants’ general ability to correctly recognize emotions when faces are masked and not masked, may have an influence on the effect produced by proposers’ (un)trustworthiness. The basic idea was that these participants might be able to gather facial information more efficiently than other when the stimulus was masked. To obtain this indicator, two possible measures were taken into consideration. The first was the difference in accuracy between the masked and unmasked conditions while, the second option was to take advantage of our GLMM on emotion recognition accuracy, which was specified with a random slope for the effect of mask for each participant. This means that for each subject, the model computes an estimate for the difference in accuracy in the masked and unmasked conditions. While these two measures are highly correlated (*r* = 0.814, *p* < 0.001), we opted for the second one because it showed a better continuous normal distribution, and it was computed within a model that considers the difficulty of different emotions. As first, we tested the role of this variable on discounting parameter *k* by reperforming the same mixed effect model, to the log-transformed *k* discounting parameter with current proposer as fixed effect and a random intercept for each subject. Using the same *t* as z criterion for significance we find similar results that is both masked untrustworthy proposer and the masked male neutral proposer elicit a significant increase in discounting rate. If we look at the back-transformed estimated values of the k parameters, we can see that although significant, the size of these effects is smaller and the increase from the baseline is much less evident (see Supplementary Table S3; Supplementary Figure S1A).

Furthermore, we also conducted the second mixed effect model for *k* parameters on the proposer conditions (without baseline), with gender and trustworthiness as fixed effects (see Supplementary Table S4). As for previous model, Omnibus Wald chi-square tests revealed no effect of emotion recognition ability, and that neither gender nor the level of trustworthiness of the proposer have a significant effect on the rate at which participants discount delayed options (Supplementary Figure S1B).

#### The Impact of Emotion Recognition Ability on Response Times

We used the same generalized mixed effect model for response times. We set the family of the distribution to inverse Gaussian and we coded the model with current proposer as fixed effect, and with a random intercept and slope for each subject. Again, similarly to study 1, we find that all proposer conditions are significantly faster than the baseline condition, but emotion recognition ability effect was not significant. A significant interaction was detected when considering emotion recognition ability with male trustworthy proposer and with male untrustworthy proposer (see Supplementary Table S5). We then plotted response times across condition (see Supplementary Figure S1C) with 5 levels of emotion recognition ability (at 10th, 25th, 50th, 75th, and 90th percentiles).

Finally, we retest effects of gender, level of trustworthiness and given response after including emotion recognition ability. Omnibus Wald tests showed a significant effect of emotion recognition ability and its interaction with the condition (see Supplementary Tables S6A, S6B). Again, we plotted response times across proposers’ trustworthiness levels with different level of emotion (Supplementary Figure S1D).

### Results Experiment B

#### Probability Discounting

**Figure 5:**
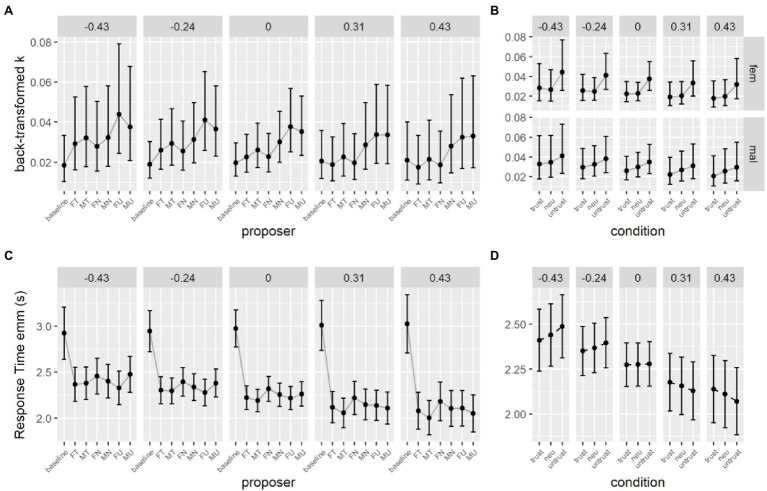
**(A)** Parameter estimates from the first model and (**B)** parameter estimates from the second model without the baseline after including emotion recognition ability. **(C)** Response times across conditions at various levels of emotion recognition ability. **(D)** Response times across proposers’ trustworthiness levels at various levels of emotion recognition ability.

Similarly to delay discounting data, a mixed effects model on log-transformed *h* parameters, with current proposer as fixed effect and a random intercept for each subject, with the baseline condition as reference level, was performed. Using the *t* as z criterion, results revealed that the untrustworthy proposers again elicited a higher discounting rate h compared to baseline. Also, the male neutral proposer is not different from baseline, while the female neutral and the male trustworthy proposers laid around the significance threshold of *t* > 2 (Table 6; Figure 5A).

**Table 6:** Fixed effects of proposer on probability discounting compared to baseline.

				CI 95%	
Term	Estimate	SE	Statistic		
				LL	UL
(Intercept)	1.222	0.129	**9.447**	0.969	1.476
Proposer FT	0.036	0.077	0.467	−0.115	0.188
Proposer MT	0.152	0.077	1.965	0.000	0.303
Proposer FN	0.157	0.077	**2.031**	0.006	0.309
Proposer MN	0.040	0.077	0.512	−0.112	0.191
Proposer FU	0.244	0.077	**3.161**	0.093	0.396
Proposer MU	0.164	0.077	**2.124**	0.013	0.316

*Significant results are in bold.*

**Figure 6:**
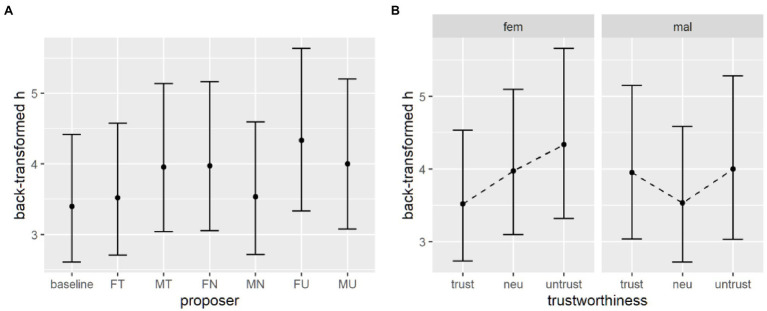
Probability discounting parameters, back-transformed from the log space. **(A)** Parameter estimates from the first model. **(B)** Parameter estimates from the second model without the baseline.

Looking at the second mixed effect model on discounting parameters, Wald tests showed that the interaction between gender and trustworthiness level was at the edge of significance. Indeed, pairwise comparisons for the three levels of trustworthiness indicated no significant difference (see Supplementary Table S2). The only significant difference was detected between the trustworthy and untrustworthy female proposers (see Table 7; Figure 6B).

**Table 7:** *Post-hoc* comparison on log(h) between levels of trustworthiness for female and male proposers.

Contrast	Gender	Estimate	SE	DF	Statistic	*p*
Trustworthy – neutral	Female	−0.121	0.066	107	−1.822	0.214
Trustworthy **–** untrustworthy	Female	−0.208	0.076	88	**−2.745**	**0.022**
Neutral – untrustworthy	Female	−0.087	0.075	89	−1.164	0.743
Trustworthy – neutral	Male	0.112	0.066	107	1.692	0.281
Trustworthy – untrustworthy	Male	−0.012	0.076	88	−0.162	>0.999
Neutral – untrustworthy	Male	−0.125	0.075	89	−1.661	0.300

*Significant results are in bold.*

#### Response Times

A generalized mixed effect model was performed to the analysis for response times in the probability discounting task, comparing the proposer conditions to the baseline. All coefficients are negative and significant (Table 8), and the estimated marginal means revealed that the baseline condition elicited indeed slower responses (Figure 6A).

**Table 8:** Fixed effects of proposer compared to baseline on RTs in the probability discounting task.

				CI 95%	
**Term**	Estimate	SE	Statistic		
				LL	UL
(Intercept)	2.691	0.114	**23.636**	2.467	2.914
Proposer FT	−0.891	0.090	**−9.889**	−1.067	−0.714
Proposer MT	−1.031	0.090	**−11.521**	−1.207	−0.856
Proposer FN	−0.942	0.087	**−10.804**	−1.113	−0.771
Proposer MN	−1.005	0.089	**−11.313**	−1.179	−0.831
Proposer FU	−0.975	0.093	**−10.442**	−1.158	−0.792
Proposer MU	−0.954	0.097	**−9.887**	−1.144	−0.765

*Significant results are in bold.*

**Figure 7:**
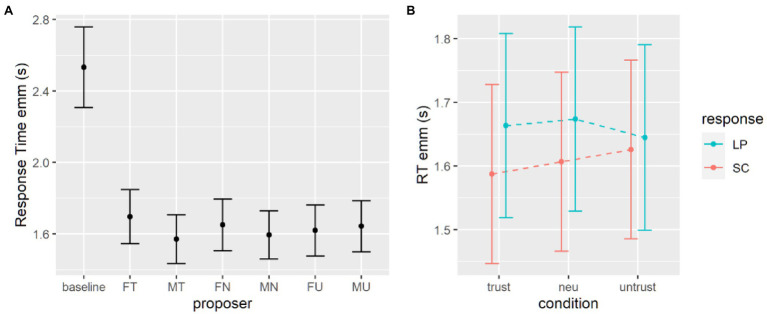
Estimates for response times in the probability discounting task. **(A)** Estimates from the first model, where the baseline is the condition with the highest RTs. **(B)** Estimates from the second model, without the baseline data. SC, small certain option; LP, later probabilistic option.

Looking into the differences within the proposer conditions, the second generalized mixed effect model’s results revealed that the only significant effect is the given response. In particular, participants were slightly faster when choosing the smaller certain option rather than the larger probabilistic one (Table 9; Figure 7B). No other main nor interaction effects were found to be significant.

**Table 9:** Omnibus test for effects of gender, trustworthiness level, and response on RTs.

	*χ* ^2^	DF	*p*
(Intercept)	**612.10**	1	**<0.001**
Gender	1.23	1	0.267
Trustworthiness level	0.51	2	0.777
Response	**8.96**	1	**0.003**
Gender: trustworthiness level	4.87	2	0.088
Gender: response	0.09	1	0.765
Trustworthiness level: response	2.09	2	0.351
Gender: trustworthiness level: response	0.59	2	0.745

*Significant results are in bold.*

#### The Role of Emotion Recognition Ability on *h* Parameter

We reperformed the mixed effects model on log-transformed *h* parameters, with current proposer as fixed effect and a random intercept for each subject, with the baseline condition as reference level, and including the participants’ random slope for emotion recognition ability with mask stimuli. As for effect on *k* parameters, we found no significant differences in the model outcomes after including emotion recognition ability (Supplementary Table S7).

Looking at the second mixed effect model on discounting parameters, Wald tests show that the interaction between gender and trustworthiness is now at the edge of significance (Supplementary Table S8). Indeed, pairwise comparisons for the three levels of trustworthiness show no significant difference. The only significant difference is between the trustworthy and untrustworthy female proposers (*t* = −2.74, *p* = 0.02). No effect of emotion recognition ability was detected.

#### The Role of Emotion Recognition Ability on Response Times

We repeated the analysis for response times in the probability discounting task, comparing the proposer conditions to the baseline with a generalized mixed effect model. All coefficients are negative and significant, and we can see from the estimated marginal means that the baseline condition is indeed still slower. As for h parameters, no main effect of emotion recognition ability neither interaction was highlighted (see Supplementary Table S9).

Looking into the differences within the proposer conditions, with the second generalized mixed effect model, we see that the only significant effect is the given response, in particular participants are slightly faster when choosing the certain option. We can also observe that no main neither interaction effect with emotion recognition ability were detected (see Supplementary Table S10).

## Discussion

In previous study, Anzani et al. (2022) reported that, when faced with an untrustworthy proposer during intertemporal and risky choice, participants modify their discounting behavior by increasing preference toward immediate and sure alternatives, thus showing a more protective choice pattern. In this study we replicated the same paradigm, trying to verify if face masks used as a safety measure against COVID-19 could influence the proposer’s perceived trustworthiness and, therefore, it modulates the impact of proposer’s perceived trustworthiness on participants’ decision-making outcomes. In particular, as based on existing evidence, which suggests that wearing a mask reduces perceived trustworthiness (Biermann et al., 2021; Gabrieli and Esposito, 2021), we may expect that the presence of the mask would let participants to perceive untrustworthy proposers as even more untrustworthy, thus amplifying the effect of proposers’ untrustworthiness on discounting behavior. On the other side, in a more indirect way, as literature suggested that wearing a mask increases perceived trustworthiness (Pandey and Zayas, 2021), we could also expect that the presence of the mask produces an increase in perceived trustworthiness for untrustworthy proposers, thus reducing the effect of proposers’ untrustworthiness on discounting behavior. To address this issue, we first looked at the differences in the discounting parameters between the baseline and the masked proposer conditions. In the delay discounting task (experiment A), untrustworthy proposers and the male neutral proposer still induced a steeper discounting compared to the baseline, but the effect is smaller when compared to the previous study with proposers’ faces full visible (Anzani et al., 2022). In the probability discounting task (experiment B), results were more surprising: even the male trustworthy masked proposer induced a higher discounting rate compared to the baseline even if the effect is not significant. In general, the estimated discounting parameters in all the seven conditions appears to be more similar to each other, also because the baseline discounting parameter appears to be higher than what reported previously (Anzani et al., 2022). Looking into the effects of the proposer’s gender and level of trustworthiness for the delay discounting task, we found no significant effect, even if plot of estimates for each condition revealed a similar pattern to what observed in Anzani et al. (2022). For the probability discounting task, a main effect of level of trustworthiness and the interaction between gender and trustworthiness were detected, both at the very edge of significance. Looking at the *post-hoc* comparisons and at the parameter estimates, results seem to suggest that only with the female proposers, the h parameter with untrustworthy proposers is significantly higher than with the trustworthy ones, while all other comparisons were not significant. Compared to discounting rates, results on response times analysis revealed a more similar patter to what reported by Anzani et al. (2022). Still, in the baseline condition participants took more time to decide compared to the masked proposer conditions, and these differences appear to be even more pronounced. Looking at the differences within the proposer conditions, the only effect that we find is still the final choice (given response), meaning that uncertain and delayed options require more time to decide, and the level of trustworthiness has little to no effect.

Results are quite the same when participant’s ability to recognize emotions from a face with mask is added to the tested models. Interestingly, the only significant effects (main or interaction with trustworthiness level) for the emotion recognition ability have been detected within experiment A (for *k* parameters and response times in delay discounting task) but those effects are not present for dependent variables in experiment B.

We consider these results to be in line with two possible explanations. As a first possibility, as face mask covers part of the proposer’s face that was a source of information in the evaluation process of their (un)trustworthiness, participants may have perceived untrustworthy faces as less untrustworthy and trustworthy faces as less trustworthy, thus producing an approximation between the effects of the two conditions. As second, due to an increase in perceived attractiveness and to a consequent increase in perceived trustworthiness, untrustworthy proposers may have been perceived as less untrustworthy. This conclusion would also been supported by evidence reporting that, in first impression creation with not masked faces, facial attractiveness evaluation precedes trustworthiness evaluation (Gutierrez-Garcia et al., 2019). This second explanation, by the way, would not justify why, in the current study, trustworthy masked proposers were possibly perceived as less trustworthy given that, in some cases, compared to results reported by Anzani et al. (2022), they produced effects that are more in line with untrustworthy proposers’ effect.

At last, when considering results on masked emotion recognition ability itself, our results seem to be in line with previously reported findings, particularly for what concerns the extremely lower accuracy on masked disgusted stimuli compared to other masked emotions (e.g., Carbon et al., 2022).

## Conclusion

Nevertheless, our conclusion may have been impacted by some methodological limits. First of all, perceived trustworthiness (trustworthy, neutral, and untrustworthy) of stimuli used as proposers was not directly evaluated by participants in this study but they were extracted from a validated database. Therefore, it was not possible to ascertain that each stimulus was indeed perceived as expected by the current sample. As second, a measure of perceived attractiveness for each presented stimulus, may have helped in disentangle the effect induced by adding a mask on proposer’s face. As third relevant limit, it would have been useful to include a measure of participants’ perception and/or beliefs about the role of mask wearing in social perception of trustworthiness as this, in addition to the occlusion of physical facial cues, may have produced the highlighted effects. Nonetheless, participants’ history with COVID-19 (both from personal and vicarious perspective) may have an influence on masked face perception and should, therefore, taken into account in future studies. Furthermore, as a general consideration to better evaluate the role of mask, it may be useful for future studies to replicate data collection by applying a within subject design for stimulus typology (with mask and without mask) as well as to include eye movements recording to help deepening our understanding of the perceptual dynamic that may contribute to discounting preferences’ change and the participant’s body state during emotion recognition task given evidence suggesting its contribution, particularly when considering disgust stimuli (e.g., Pezzulo et al., 2018). At last, as future direction, it would be particularly useful to replicate the experimental protocol by controlling for ethnic matching between face stimuli and the respondent as well as with elderly participants given the well-known positivity effect (e.g., Di Domenico et al., 2015) that may affect their ability to perceive facial (un)trustworthiness (e.g., Chen et al., 2021) as well as because they represent a risk population for fraud (Burnes et al., 2017).

## Data Availability Statement

The datasets presented in this study can be found in online repositories. The names of the repository/repositories and accession number(s) can be found at: https://osf.io/qu8fg/view_only=d2307c78bbd64b4ea782f822f3314a43.

## Ethics Statement

The studies involving human participants were reviewed and approved by Institutional Review Board of Psychology (IRBP) of the Department of Psychological, Health and Territorial Sciences at G. d Annunzio University of Chieti-Pescara. The patients/participants provided their written informed consent to participate in this study.

## Author Contributions

LC conceived the experiment. SA and AB prepared tasks and conducted the experiment. LC and SA performed statistical analysis, figure generation, and prepared the draft manuscript. All authors contributed to the article and approved the submitted version.

## Conflict of Interest

The authors declare that the research was conducted in the absence of any commercial or financial relationships that could be construed as a potential conflict of interest.

## Publisher’s Note

All claims expressed in this article are solely those of the authors and do not necessarily represent those of their affiliated organizations, or those of the publisher, the editors and the reviewers. Any product that may be evaluated in this article, or claim that may be made by its manufacturer, is not guaranteed or endorsed by the publisher.

## Acknowledgments

We wish to thank all the participants and Lucia Casciano for helping in data gathering.

## Supplementary Material

The Supplementary Material for this article can be found online at: https://www.frontiersin.org/articles/10.3389/fpsyg.2022.926520/full#supplementary-material

## Footnotes

^1^ https://support.pstnet.com/
