# Supplementary material for: Face Mask Reduces the Effect of Proposer’s (Un)Trustworthiness on Intertemporal and Risky Choices
Source: Front Psychol. 2022 Jun 16;13:926520. doi: 10.3389/fpsyg.2022.926520 (PMC9243543; doi:10.3389/fpsyg.2022.926520)

Supplementary Material

# Supplementary Tables

# Table S1. Omnibus test for the effects of gender and trustworthiness on k parameters. Significant effects in bold.

|  | **χ²** | **DF** | ***p*** |
| --- | --- | --- | --- |
| **(Intercept)** | **437.69** | 1 | **<0.001** |
| Trustworthiness Level | 3.74 | 2 | 0.154 |
| Gender | 2.39 | 1 | 0.122 |
| Trustworthiness Level: Gender | 4.93 | 2 | 0.085 |

# Table S2. Omnibus test of effects of gender and trustworthiness on probability discounting parameters with masked proposers. Significant results are in bold.

|  | **χ²** | **DF** | ***p*** |
| --- | --- | --- | --- |
| **(Intercept)** | **127.44** | 1 | **<0.001** |
| Gender | 0.47 | 1 | 0.495 |
| Trustworthiness Level | 3.98 | 2 | 0.137 |
| **Gender: Trustworthiness Level** | **7.14** | **2** | **0.028** |

# Table S3. Fixed effects of proposers compared to baseline. Significant results are in bold.

|  | | | | | | **CI 95%** | |
| --- | --- | --- | --- | --- | --- | --- | --- |
| **Term** | **Estimate** | | | **SE** | **Statistic** | **LL** | **UL** |
| **(Intercept)** | -3.930 | | | 0.206 | **-19.038** | -4.335 | -3.526 |
| Proposer FT | 0.137 | | | 0.181 | 0.758 | -0.217 | 0.491 |
| Proposer MT | 0.287 | | | 0.181 | 1.589 | -0.067 | 0.641 |
| Proposer FN | 0.147 | | | 0.181 | 0.814 | -0.207 | 0.501 |
| **Proposer MN** | | 0.426 | 0.181 | | **2.357** | 0.072 | 0.780 |
| **Proposer FU** | | 0.652 | 0.181 | | **3.608** | 0.298 | 1.006 |
| **Proposer MU** | | 0.583 | 0.181 | | **3.227** | 0.229 | 0.937 |
| Emotion Recognition Ability | | 0.145 | 0.544 | | 0.266 | -0.922 | 1.211 |
| Proposer FT: Emotion Recognition Ability | | -0.743 | 0.476 | | -1.560 | -1.676 | 0.190 |
| Proposer MT: Emotion Recognition Ability | | -0.617 | 0.476 | | -1.296 | -1.550 | 0.316 |
| Proposer FN: Emotion Recognition Ability | | -0.619 | 0.476 | | -1.299 | -1.552 | 0.315 |
| Proposer MN: Emotion Recognition Ability | | -0.307 | 0.476 | | -0.644 | -1.240 | 0.626 |
| Proposer FU: Emotion Recognition Ability | | -0.500 | 0.476 | | -1.049 | -1.433 | 0.434 |
| Proposer MU: Emotion Recognition Ability | | -0.299 | 0.476 | | -0.627 | -1.232 | 0.635 |

#

# Table S4. Omnibus test for the effects of gender and trustworthiness on k parameters.

|  | **χ²** | **DF** | **p** |
| --- | --- | --- | --- |
| **(Intercept)** | **433.90** | 1 | **<0.001** |
| Trustworthiness Level | 3.67 | 2 | 0.160 |
| Gender | 2.40 | 1 | 0.121 |
| Emotion Recognition Ability | 0.90 | 1 | 0.343 |
| Trustworthiness Level: Gender | 4.91 | 2 | 0.086 |
| Trustworthiness Level: Emotion Recognition Ability | 0.71 | 2 | 0.701 |

# Table S5. Fixed effects of proposers compared to baseline and emotion recognition ability on RTs in the delay discounting task. Significant results are in bold.

|  | | | | **CI 95%** | |
| --- | --- | --- | --- | --- | --- |
| **Term** | **Estimate** | **SE** | **Statistic** | **LL** | **UL** |
| **(Intercept)** | 2.974 | 0.102 | **29.015** | 2.773 | 3.175 |
| **Proposer FT** | -0.751 | 0.103 | **-7.314** | -0.952 | -0.549 |
| **Proposer MT** | -0.782 | 0.106 | **-7.360** | -0.991 | -0.574 |
| **Proposer FN** | -0.655 | 0.098 | **-6.669** | -0.847 | -0.462 |
| **Proposer MN** | -0.719 | 0.099 | **-7.252** | -0.914 | -0.525 |
| **Proposer FU** | -0.756 | 0.096 | **-7.879** | -0.944 | -0.568 |
| **Proposer MU** | -0.711 | 0.105 | **-6.798** | -0.916 | -0.506 |
| Emotion Recognition Ability | 0.118 | 0.265 | 0.445 | -0.402 | 0.638 |
| Proposer FT: Emotion Recognition Ability | -0.453 | 0.263 | -1.720 | -0.969 | 0.063 |
| **Proposer MT: Emotion Recognition Ability** | -0.555 | 0.274 | **-2.028** | -1.092 | -0.019 |
| Proposer FN: Emotion Recognition Ability | -0.438 | 0.251 | -1.747 | -0.930 | 0.053 |
| Proposer MN: Emotion Recognition Ability | -0.463 | 0.254 | -1.823 | -0.961 | 0.035 |
| Proposer FU: Emotion Recognition Ability | -0.373 | 0.245 | -1.524 | -0.854 | 0.107 |
| **Proposer MU:** **Emotion Recognition Ability** | -0.612 | 0.270 | **-2.269** | -1.140 | -0.083 |

# Table S6A. Omnibus test of effects for gender, trustworthiness level, emotion recognition ability and given response on RTs.

|  | **χ²** | **DF** | **p** |
| --- | --- | --- | --- |
| **(Intercept)** | **1490.64** | 1 | **<0.001** |
| Gender | 0.08 | 1 | 0.772 |
| Trustworthiness Levels | 0.02 | 2 | 0.990 |
| **Response** | **57.51** | 1 | **<0.001** |
| **Emotion Recognition Ability** | **6.53** | 1 | **0.011** |
| Gender: Trustworthiness Levels | 3.19 | 2 | 0.203 |
| Gender: Response | 0.10 | 1 | 0.751 |
| Trustworthiness Levels: Response | 5.70 | 2 | 0.058 |
| **Trustworthiness Levels:** **Emotion Recognition Ability** | **8.52** | 2 | **0.014** |
| Gender: Trustworthiness Levels: Response | 0.36 | 2 | 0.835 |

# Table S6B. Bonferroni corrected comparisons on post-hoc interaction between trustworthiness level and emotion recognition ability as covariate.

| **Contrast** | **Estimate** | **SE** | **DF** | **z.ratio** | ***p*** |
| --- | --- | --- | --- | --- | --- |
| Trustworthy - Neutral | 0.066 | 0.059 | Inf | 1.125 | 0.782 |
| **Trustworthy - Untrustworthy** | **0.168** | **0.058** | **Inf** | **2.907** | **0.011** |
| Neutral - Untrustworthy | 0.101 | 0.060 | Inf | 1.683 | 0.277 |

# Table S7. Fixed effects of proposers on probability discounting compared to baseline. Significant results are in bold.

|  | | | | **CI 95%** | |
| --- | --- | --- | --- | --- | --- |
| **Term** | **Estimate** | **SE** | **Statistic** | **LL** | **UL** |
| **(Intercept)** | 1.226 | 0.134 | **9.180** | 0.964 | 1.487 |
| Proposer FT | 0.035 | 0.080 | 0.439 | -0.121 | 0.191 |
| Proposer MT | 0.149 | 0.080 | 1.870 | -0.007 | 0.305 |
| Proposer FN | 0.149 | 0.080 | 1.874 | -0.007 | 0.305 |
| Proposer MN | 0.028 | 0.080 | 0.356 | -0.128 | 0.184 |
| **Proposer FU** | 0.232 | 0.080 | **2.913** | 0.076 | 0.388 |
| Proposer MU | 0.154 | 0.080 | 1.930 | -0.002 | 0.310 |
| Emotion Recognition Ability | -0.048 | 0.377 | -0.127 | -0.787 | 0.691 |
| Proposer FT: Emotion Recognition Ability | 0.016 | 0.225 | 0.073 | -0.424 | 0.457 |
| Proposer MT: Emotion Recognition Ability | 0.044 | 0.225 | 0.195 | -0.397 | 0.485 |
| Proposer FN: Emotion Recognition Ability | 0.114 | 0.225 | 0.505 | -0.327 | 0.554 |
| Proposer MN: Emotion Recognition Ability | 0.162 | 0.225 | 0.722 | -0.278 | 0.603 |
| Proposer FU: Emotion Recognition Ability | 0.181 | 0.225 | 0.807 | -0.259 | 0.622 |
| Proposer MU: Emotion Recognition Ability | 0.153 | 0.225 | 0.679 | -0.288 | 0.593 |

#

# Table S8. Omnibus test of effects of gender and trustworthiness on probability discounting parameters with masked proposers. Significant results are in bold.

|  | **χ²** | **df** | **p** |
| --- | --- | --- | --- |
| **(Intercept)** | **119.11** | 1 | **<0.001** |
| Gender | 0.48 | 1 | 0.486 |
| Trustworthiness Level | 3.41 | 2 | 0.182 |
| **Gender: Trustworthiness Level** | **6.98** | 2 | **0.030** |
| Trustworthiness Level: Emotion Recognition Ability | 0.92 | 3 | 0.820 |

# Table S9. Fixed effects of masked proposer compared to baseline on RTs in the probability discounting task. Significant results are in bold.

|  | | | | **CI 95%** | |
| --- | --- | --- | --- | --- | --- |
| **Term** | **Estimate** | **SE** | **Statistic** | **LL** | **UL** |
| **(Intercept)** | 2.512 | 0.117 | **21.462** | 2.283 | 2.742 |
| **Proposer FT** | -0.839 | 0.091 | **-9.269** | -1.017 | -0.662 |
| **Proposer MT** | -0.951 | 0.090 | **-10.614** | -1.127 | -0.775 |
| **Proposer FN** | -0.876 | 0.087 | **-10.017** | -1.047 | -0.704 |
| **Proposer MN** | -0.936 | 0.090 | **-10.442** | -1.112 | -0.760 |
| **Proposer FU** | -0.911 | 0.094 | **-9.694** | -1.095 | -0.727 |
| **Proposer MU** | -0.889 | 0.097 | **-9.163** | -1.079 | -0.699 |
| Emotion Recognition Ability | 0.302 | 0.337 | 0.898 | -0.358 | 0.962 |
| Proposer FT: Emotion Recognition Ability | 0.019 | 0.265 | 0.071 | -0.500 | 0.537 |
| Proposer MT: Emotion Recognition Ability | -0.160 | 0.261 | -0.615 | -0.672 | 0.351 |
| Proposer FN: Emotion Recognition Ability | -0.117 | 0.255 | -0.459 | -0.616 | 0.382 |
| Proposer MN: Emotion Recognition Ability | -0.052 | 0.261 | -0.197 | -0.564 | 0.461 |
| Proposer FU: Emotion Recognition Ability | -0.054 | 0.274 | -0.197 | -0.591 | 0.483 |
| Proposer MU: Emotion Recognition Ability | -0.033 | 0.284 | -0.118 | -0.589 | 0.522 |

# Table S10. Omnibus test of effects for gender, trustworthiness and given response on RTs.

|  | **χ²** | **DF** | **p** |
| --- | --- | --- | --- |
| **(Intercept)** | **510.61** | 1 | **<0.001** |
| Gender | 1.24 | 1 | 0.265 |
| Trustworthiness Level | 0.49 | 2 | 0.783 |
| **Response** | **8.83** | 1 | **0.003** |
| Emotion Recognition Ability | 1.26 | 1 | 0.261 |
| Gender: Trustworthiness Level | 4.99 | 2 | 0.083 |
| Gender: Response | 0.09 | 1 | 0.769 |
| Trustworthiness Level: Response | 2.12 | 2 | 0.346 |
| Trustworthiness Level: Emotion Recognition Ability | 0.07 | 2 | 0.964 |
| Gender: Trustworthiness Level: Response | 0.61 | 2 | 0.736 |

# Figure S1. A) Parameter estimates from the first model and B) Parameter estimates from the second model without the baseline after including emotion recognition ability. C) Response times across conditions at various levels of emotion recognition ability. D) Response times across proposers’ trustworthiness levels at various levels of emotion recognition ability.

#
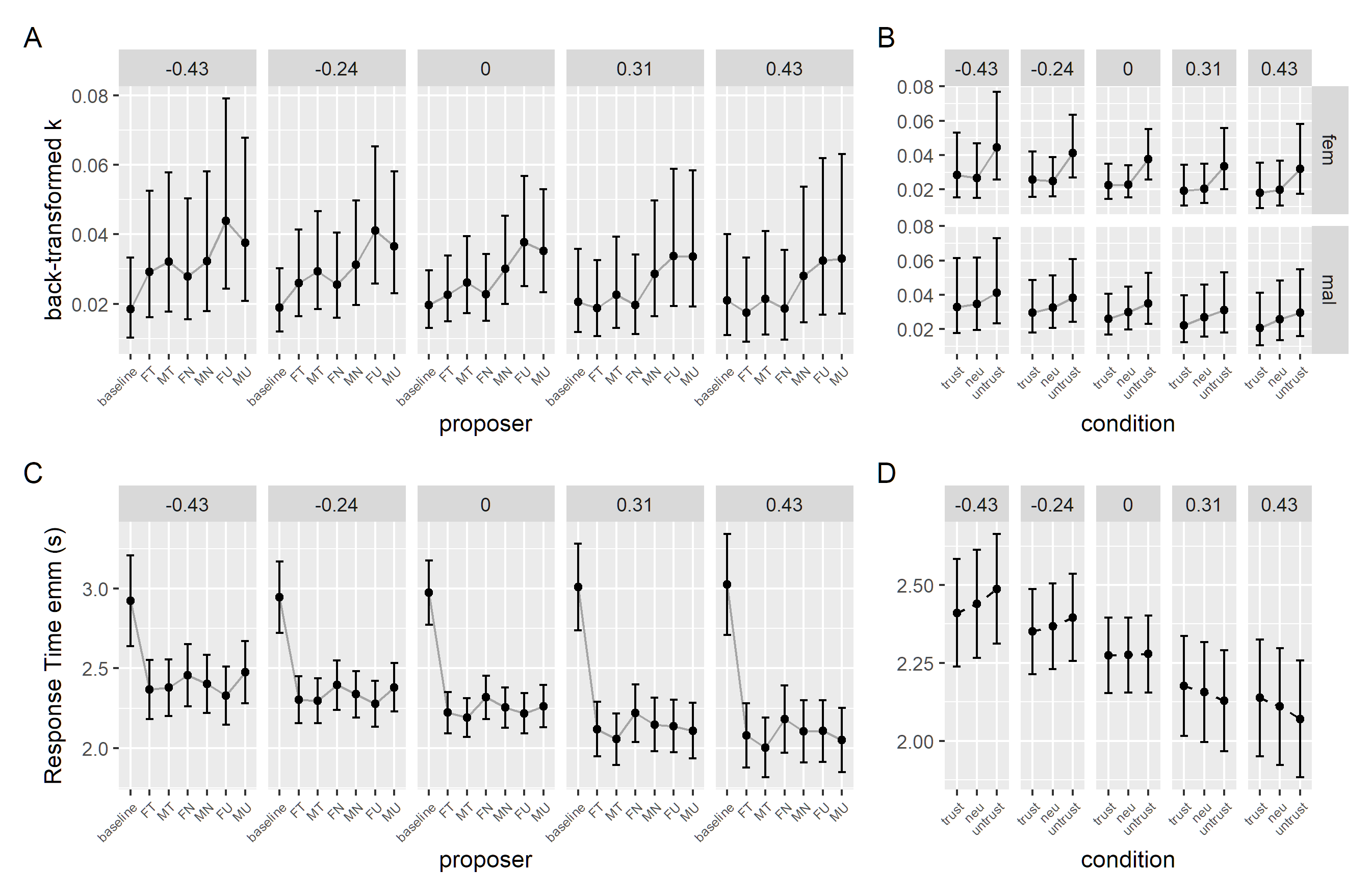

Supplement: Supplementary file 1 [file Table_1.DOCX]
